# α-Synuclein facilitates endocytosis by elevating the steady-state levels of phosphatidylinositol 4,5-bisphosphate

**DOI:** 10.1074/jbc.RA120.015319

**Published:** 2021-01-13

**Authors:** Meir Schechter, Merav Atias, Suaad Abd Elhadi, Dana Davidi, Daniel Gitler, Ronit Sharon

**Affiliations:** 1 Department of Biochemistry and Molecular Biology, IMRIC, The Hebrew University-Hadassah Medical School, Jerusalem, Israel; 2 Department of Physiology and Cell Biology, Faculty of Health Sciences, Ben-Gurion University of the Negev, Beer-Sheva, Israel; 3 Zlotowski Center for Neuroscience, Ben-Gurion University of the Negev, Beer-Sheva, Israel

**Keywords:** Clathrin-mediated endocytosis, phosphoinositides, synaptic vesicles cycling, phosphorylated AP2, α-Synuclein, alpha-synuclein (α-synuclein), phosphoinositide, clathrin, endocytosis, synapse

## Abstract

α-Synuclein (α-Syn) is a protein implicated in the pathogenesis of Parkinson's disease (PD). It is an intrinsically disordered protein that binds acidic phospholipids. Growing evidence supports a role for α-Syn in membrane trafficking, including, mechanisms of endocytosis and exocytosis, although the exact role of α-Syn in these mechanisms is currently unclear. Here we investigate the associations of α-Syn with the acidic phosphoinositides (PIPs), phosphatidylinositol 4,5-bisphosphate (PI(4,5)P_2_) and phosphatidylinositol 3,4-bisphosphate (PI(3,4)P_2_). Our results show that α-Syn colocalizes with PIP_2_ and the phosphorylated active form of the clathrin adaptor protein 2 (AP2) at clathrin-coated pits. Using endocytosis of transferrin as an indicator for clathrin-mediated endocytosis (CME), we find that α-Syn involvement in endocytosis is specifically mediated through PI(4,5)P_2_ levels on the plasma membrane. In accord with their effects on PI(4,5)P_2_ levels, the PD associated A30P, E46K, and A53T mutations in α-Syn further enhance CME in neuronal and nonneuronal cells. However, lysine to glutamic acid substitutions at the KTKEGV repeat domain of α-Syn, which interfere with phospholipid binding, are ineffective in enhancing CME. We further show that the rate of synaptic vesicle (SV) endocytosis is differentially affected by the α-Syn mutations and associates with their effects on PI(4,5)P_2_ levels, however, with the exception of the A30P mutation. This study provides evidence for a critical involvement of PIPs in α-Syn–mediated membrane trafficking.

α-Synuclein (α-Syn) protein is critically implicated in the pathogenesis of Parkinson's disease (PD). α-Syn reversibly interacts with membrane lipids. It has been shown to preferentially bind acidic phospholipids, including, phosphatidic acid (PA), phosphatidylserine (PS), and phosphatidylinositol (PI) (1, 2, 3), and the acidic phosphoinositides (PIPs) (4, 5, 6, 7). α-Syn also interacts with fatty acids (8, 9, 10). Upon lipid binding, the intrinsically disordered α-Syn protein acquires an α-helical structure (1).

α-Syn plays a role in membrane trafficking and synaptic vesicle cycling (11), however, its exact role in these mechanisms is far from being clear. In a previous study, we reported the first indication for a role of α-Syn in clathrin-mediated endocytosis (CME) and synaptic vesicle (SV) cycling (12). We suggested that α-Syn acts to increase membrane curvature through enrichment of membrane phospholipids with polyunsaturated fatty acids (PUFAs) and increased membrane fluidity (9, 12). It was further suggested based on α-, β-, γ-Syn knock-out in mice that all three synucleins are involved in clathrin-mediated SV recycling at presynaptic nerve terminals (13).

Side by side with studies reporting an activating role, other studies reported that excess of α-Syn in the synapse interferes with endocytosis (4, 14, 15). α-Syn was shown to inhibit SV endocytosis during intense electrical stimulation of lamprey neurons (16). Furthermore, neurons overexpressing α-Syn internalized lower amounts of styryl dyes, which serve as indicators for SV recycling, suggesting a reduction in endocytosis (17, 18).

In addition to its reported role(s) in endocytosis, a large body of evidence indicates a role for α-Syn in mechanisms of exocytosis. These include the soluble NSF attachment proteins receptor (SNARE) complex assembly (19, 20, 21) or SNARE protein binding (22) and vesicle fusion (23, 24, 25), transmitter release (26, 27), and the regulation of fusion pore dilation (28). However, a certain degree of controversy regarding the exact role for α-Syn in exocytosis persists.

The most common PIP form in the plasma membrane is PI(4,5)P_2_, which among other cellular functions, regulates mechanisms of endocytosis and exocytosis. In endocytosis, PI(4,5)P_2_ is involved in the early steps of recruiting endocytic clathrin adaptors and their accessory factors to the plasma membrane (recently reviewed in Refs. 29 and 30). PI(4,5)P_2_ is also required for vesicle fission (31) and uncoating (32, 33). Additional PIPs are involved in CME, including PI(3,4)P_2_, which facilitates vesicle maturation and completion of the process (34, 35). In exocytosis, PI(4,5)P_2_ is involved in priming and fusion steps of Ca^2+^-triggered vesicle release (36). PI(4,5)P_2_ recruits and activates specific proteins that regulate SNARE complex assembly and function (36, 37). Additional roles for PI(4,5)P_2_ in dilation of the fusion pore have been described (36, 37). These may involve PI(4,5)P_2_-binding proteins such as CAPS and synaptotagmin (38, 39, 40) or its effects on F-actin polymerization (41).

In this study, we report evidence indicating that α-Syn's involvement in mechanisms of transferrin and SV endocytosis is specifically mediated through its activity to enrich the plasma membrane with PI(4,5)P_2_ and PI(3,4)P_2_ (PIP_2_). Our results point at PIP_2_ as key components in α-Syn–mediated mechanisms of membrane trafficking, in neuronal and nonneuronal cells.

## Results

### α*-Syn colocalizes with phosphorylated AP2 (pAP2) and PIP_2_ on clathrin-coated pits (CCPs)*

**Figure 1:**
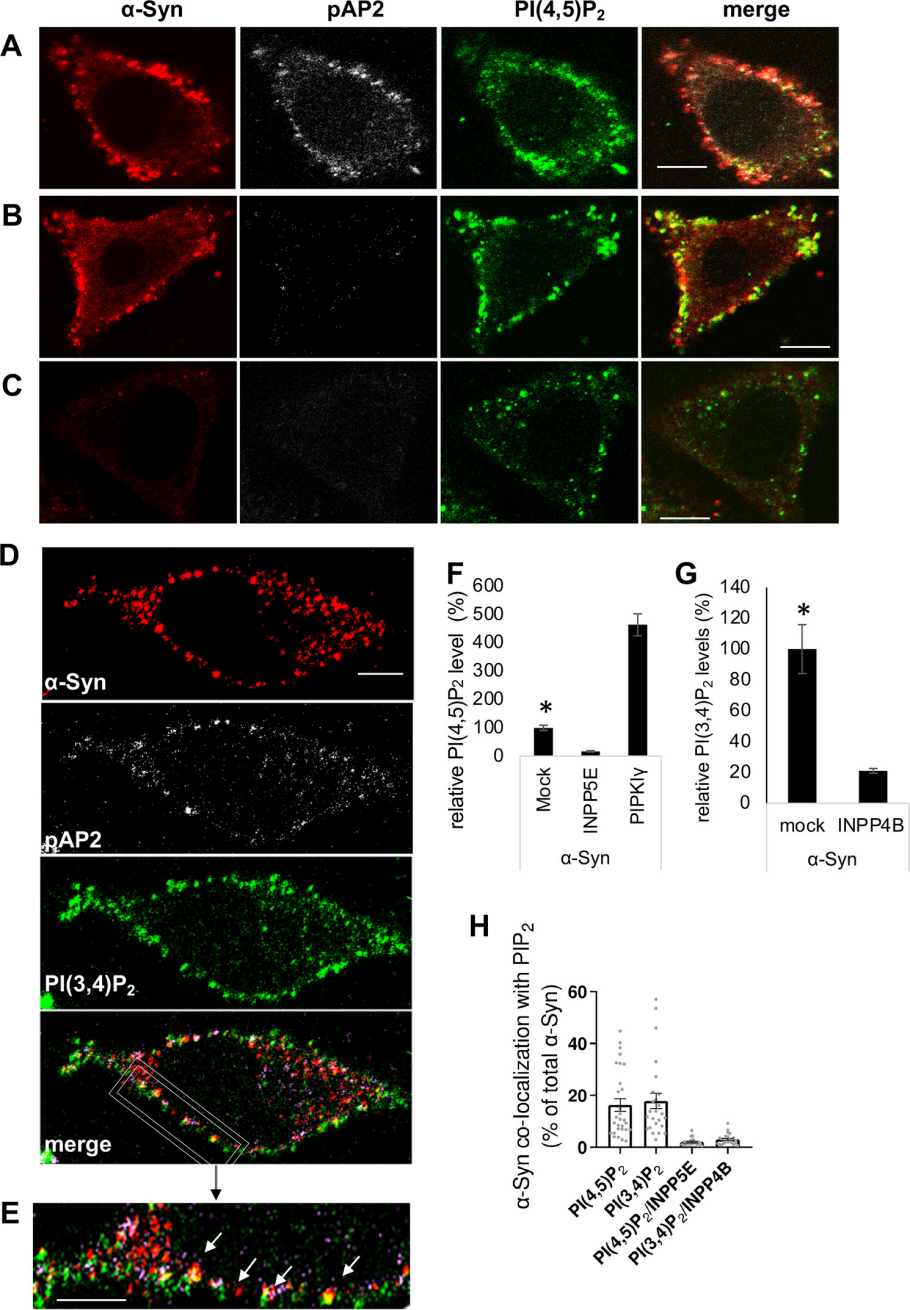
α**-Syn colocalizes with phosphorylated AP2 and PIP_2_ on clathrin-coated pits.***A,* SK-Mel2 cells were processed for the detection of the immunoreactive signals of endogenous α-Syn (ab 21976 ab, *red*), phosphorylated Thr-156 µ2 subunit of AP2 (pAP2; *gray*) and PI(4,5)P_2_ (*green*) by ICC. *Bar* = 10 μm. *B,* SK-Mel2 cells treated with LP-935509 (10 μm, for 3 h), to inhibit the phosphorylation of the µ2 subunit of AP2 and processed for ICC as in *A*. *Bar* = 10 μm. *C,* SK-Mel2 cells infected with lentivirus encoding shSNCA to silence α-Syn expression. Cells were treated with LP-935509 inhibitor as in *B* and processed for ICC. *Bar* = 10 μm. *D,* SK-Mel2 cells as in *A*, however, immunoreacted for PI(3,4)P_2_ detection (*green*). *Bar* = 5 μm. *E,* higher magnification of the image shown in *D*, focusing on plasma membrane. *Arrows* indicate spots of colocalization for α-Syn/pAP2/PI(3,4)P_2_. *Bar* = 5 μm. *F,* HEK293T cells, transfected to express the specified PIP-metabolizing proteins. Cells were analyzed by FACS to immunodetect PI(4,5)P_2_ levels. *n* > 2000 cells in each group; mean ± S.E. *, *p* < 0.01, *t* test. *G,* cells were analyzed by FACS as in *F* to detect the PI(3,4)P_2_ signal. *n* > 2000 cells; mean ± S.E. *, *p* < 0.01, *t* test. *H*, SK-Mel2 cells processed for ICC as in *A* and co-immunoreacted with anti-α-Syn (MJFR1) and anti-PIP abs (Echelon). Colocalization of the signal obtained for α-Syn with the specified PIP was quantified and normalized to total α-Syn–positive spots. Colocalization is reduced in controls cells that express the specific PIP-phosphatase. *n* > 22 cells; mean ± S.E.

Phosphorylation at Thr-156 of the μ2 subunit of the clathrin adaptor AP2 starts following its binding to PI(4,5)P_2_ at the initiation of a CCP and throughout vesicle lifetime (42, 43, 44). We analyzed SK-Mel2 cells, which express detectable levels of endogenous α-Syn protein. The immunoreactive signal for pAP2, observed by ICC, appeared on the plasma membrane of the cells. This signal colocalized with the signal obtained for α-Syn, using anti-α-Syn antibody (ab21976, Fig. 1*A*). To assess the specificity of pAP2 signal we utilized a specific inhibitor (LP-935509) for Numb-associated kinase, which phosphorylate the μ2 subunit of AP2 (42, 43, 44, 45). pAP2 signal was dramatically reduced in cells treated with the LP-935509 inhibitor (10 μm for 3 h) and no obvious colocalization of α-Syn and pAP2 could be detected (Fig. 1*B*). The specificity of the α-Syn signal was confirmed in cells that their α-Syn expression was silenced with shSNCA and treated with the LP-935509 inhibitor. The results show a substantial loss of both signals, α-Syn and pAP2 (Fig. 1*C*).

To confirm that α-Syn colocalizes with pAP2 on CCP, the slides were immunoreacted also with antibodies against PI(4,5)P_2_ (Fig. 1, *A–C*) or PI(3,4)P_2_ (Fig. 1*D* and *E*). The results show a high degree of colocalization for the immunoreactive signals obtained for α-Syn, pAP2, and either PI(4,5)P_2_ or PI(3,4)P_2_ (Fig. 1, *A–E*).

The specificity of PI(4,5)P_2_ and PI(3,4)P_2_ signals was assessed in control HEK293T cells, transfected to express the inositol polyphosphate-5-phosphatase E (INPP5E), which dephosphorylate the 5-phosphate of PI(4,5)P_2_; the inositol polyphosphate-4-phosphatase B (INPP4B), which dephosphorylate the 4-phosphate of PI(3,4)P_2_; or type Iγ PI4P-5-kinase (PIPKIγ), which produces PI(4,5)P_2_. Importantly, the PI(4,5)P_2_ signal was dramatically lower in cells expressing the INPP5E phosphatase, and ∼4.5-fold higher with the expression of PIPKIγ (Fig. 1*F*). In accord, PI(3,4)P_2_ signal was dramatically lower with the expression of INPP4B phosphatase (Fig. 1*G*).

Using a program-based method, we scanned the ICC images obtained for the SK-Mel2 cells to identify positive pixels in each channel and the colocalizing pixels within the channels. A portion of α-Syn signal specifically colocalized with PI(4,5)P_2_ (∼16%) and PI(3,4)P_2_ (∼18%). Colocalization of α-Syn with PI(4,5)P_2_ or PI(3,4)P_2_ was diminished following the expression of INPP5E or INPP4B, respectively, confirming the specificity of the results (Fig. 1*H*).

**Figure 2:**
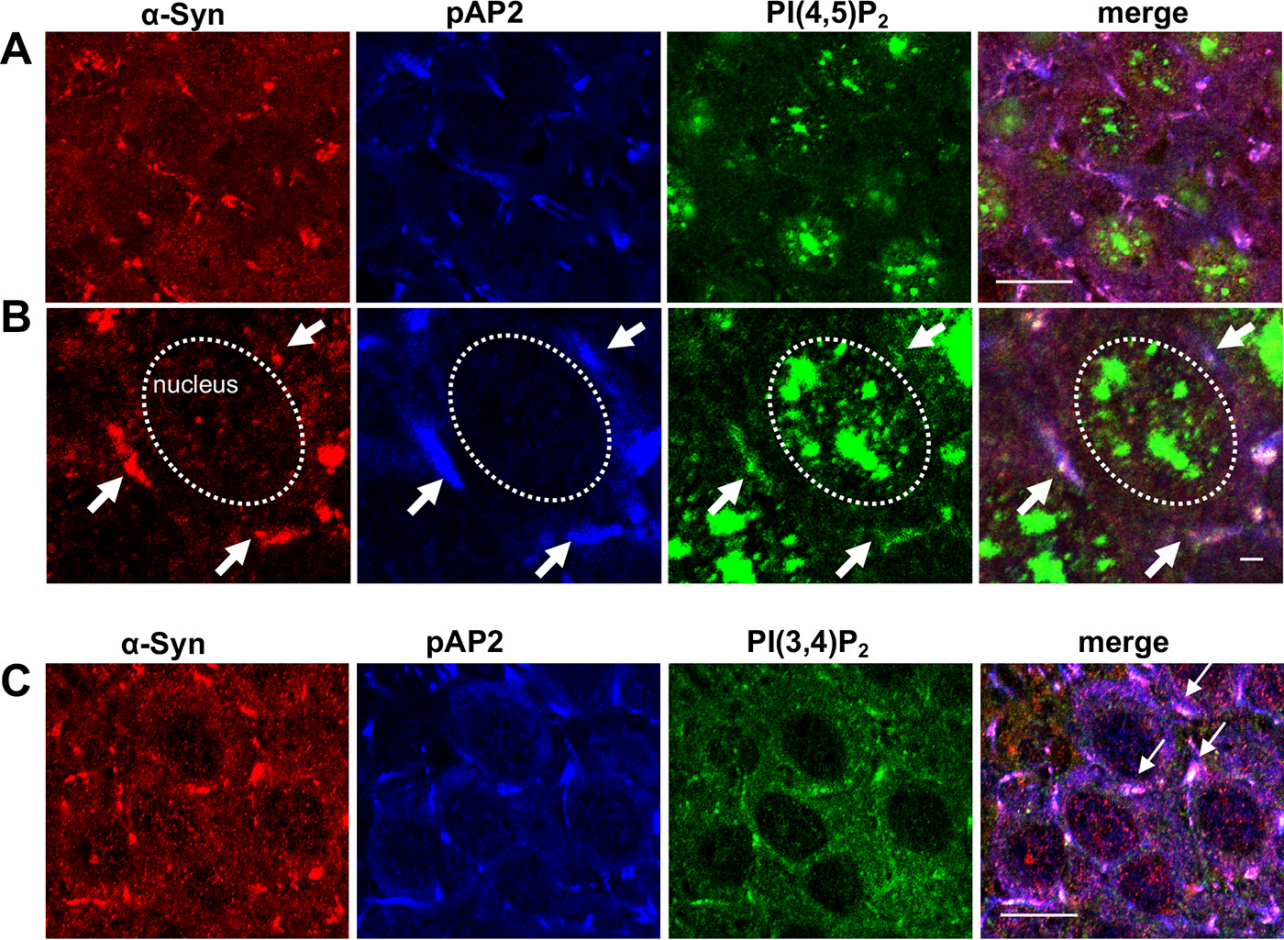
α**-Syn and PIP_2_ at CCP in mouse brains.***A,* paraffin-embedded section (6 μm) containing the hippocampus of a 2-month–old A53T α-Syn tg mouse, immunoreacted with anti-α-Syn ab (ab21976, *red*), phosphorylated Thr-156 µ2 subunit of AP2 (pAP2; *blue*) and PI(4,5)P_2_ (*green*). *Bar* = 20 μm. *B,* magnification of the image in *A* to demonstrate the colocalization between α-Syn (*red*), pAP2 (*blue*), and PI(4,5)P_2_ (*green*) surrounding the cell nucleus. Nuclei are demonstrated by the *dotted ovals*. *Bar* = 2 μm. *C,* paraffin-embedded section (6 μm) of a 2-month–old A53T α-Syn tg mouse (as in *A*), immunoreacted with anti-α-Syn (ab21976, *red*), phosphorylated Thr-156 µ2 subunit of AP2 (pAP2; *blue*) and PI(3,4)P_2_ (*green*). *Bar* = 20 μm.

We next validated the results indicating colocalization for the immunoreactive signals obtained for α-Syn, pAP2, and PI(4,5)P_2_ in coronal brain sections from an A53T α-Syn tg mouse by immunohistochemistry. The results show a strong nuclear signal with the PI(4,5)P_2_ antibody (46). In addition, colocalization between α-Syn, pAP2, and PI(4,5)P_2_ is detected surrounding the cells in the hippocampus (Fig. 2, *A* and *B*), as well as additional brain regions. Similar results were obtained in a similar set-up, were PI(3,4)P_2_ ab replaced by PI(4,5)P_2_ ab (Fig. 2*C*). Although nuclear PI(3,4)P_2_ signal is substantially weaker.

These results suggest that endogenous α-Syn localizes, at least in part, to PI(4,5)P_2_/PI(3,4)P_2_-positive endocytic CCPs, consistent with a possible function in CME.

### α*-Syn involvement in endocytosis of transferrin associates with alterations in cellular PIP_2_ levels*

**Figure 3:**
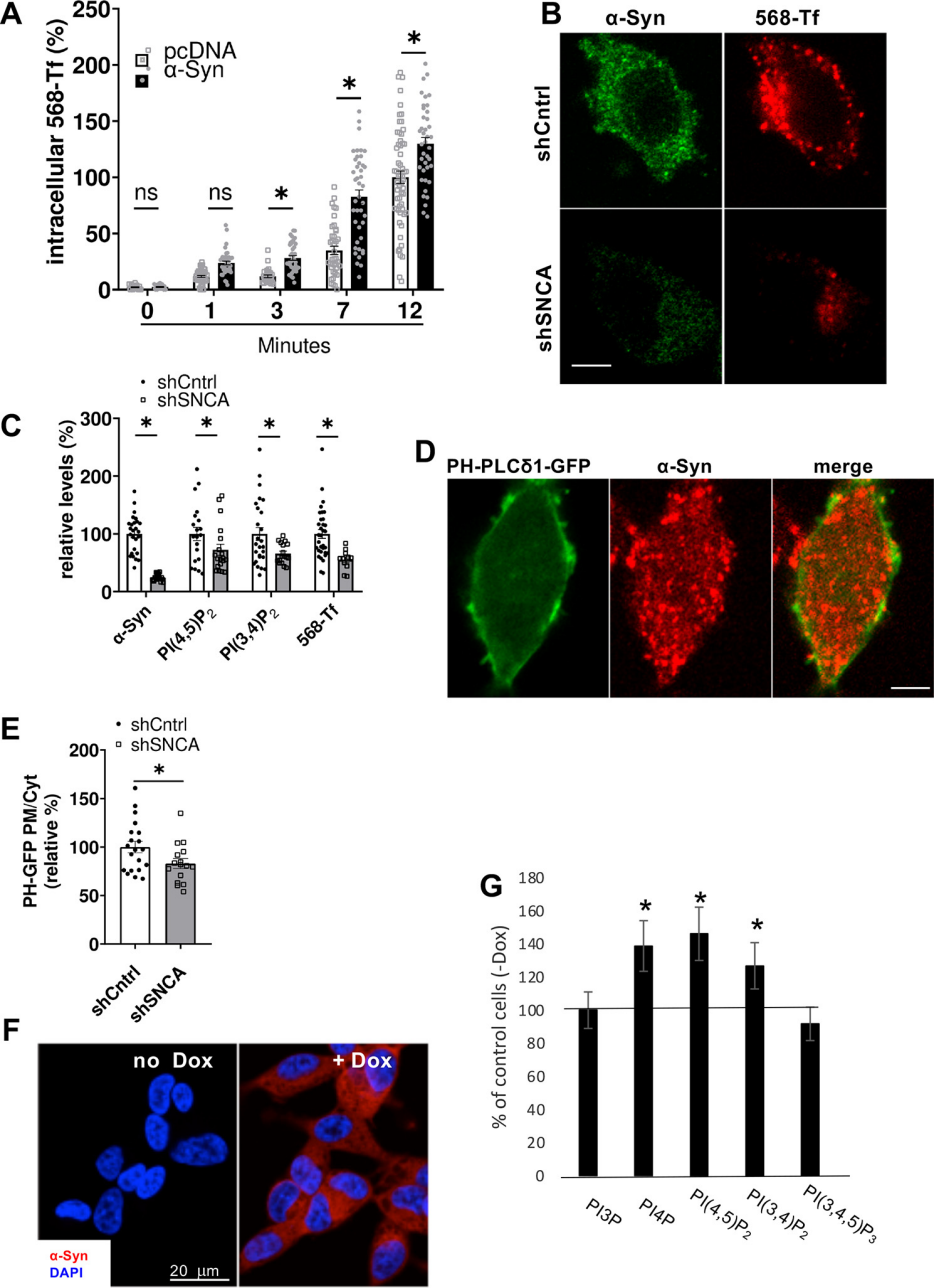
α**-Syn increases PIP_2_ levels and endocytosis of 568-Tf.***A,* the kinetics of Alexa 568-transferrin (568-Tf; 25 µg/ml) endocytosis in α-Syn or mock transfected HEK293T cells. Cells were incubated in parallel with 568-Tf for 0-12 min at 37 °C. The graph presents the sum of signal in endocytic vesicles (above threshold). *n* > 30 cell; mean ± S.E. of α-Syn (*circles*) and mock (*squares*) expressing cells. *, *p* < 0.05; *t* test with Bonferroni correction for multiple comparisons. *B,* SK-Mel2 cells infected with lentivirus encoding shSNCA or shCntrl. 568-Tf was applied to cells for 7 min and cells were processed for the detection of 568-Tf (*red*) endocytosis as in *A*. α-Syn detected with an anti-α-Syn ab (Syn211; *green*). *Bar* = 10 μm. *C,* SK-Mel2 cells expressing shSNCA or shCntrl (as in *A*). The immunoreactive signals for α-Syn, PI(4,5)P_2_, and PI(3,4)P_2_ were detected by ICC; 568-Tf was detected as in *B*. Mean ± S.E. of *n* = 17-33 cells per treatment; *, *p* < 0.05 *t* test, with Bonferroni correction for multiple comparisons. *D*, cells expressing PH-PLCδ1-GFP (*green*, direct fluorescence) and immunoreacted with anti-α-Syn ab (Syn211; *red*). *Bar* = 10 μm. *E,* the PH-PLCδ1-GFP signal ratio (plasma membrane to cytosol) determined in SK-Mel2 cells expressing shCntrl or shSNCA (*n* > 15 cells per treatment, mean ± S.E.; *p* < 0.05 *t* test). *F,* inducible, Tet-on SH-SY5Y α-Syn-expressing cells incubated with doxycycline (1 µg/ml) for 72 h or with the DMSO solvent. Cells were processed for ICC and immunoreacted with anti-α-Syn ab (MJFR1; *red*). DAPI staining depicts nuclei (*blue*). *Bars* = 20 μm. *G,* α-Syn expression was induced with doxycycline (1 µg/ml) for 72 h in the inducible SH-SY5Y cells. Control cells were treated in parallel with the solvent. Cells were immunoreacted with anti-PIP ab (Echelon) as specified, and analyzed by FACS. Results are presented as percent of control cells, with control mock-vector, set at 100%. *n* > 2000 cells per treatment; mean ± S.E.; *, *p* < 0.05; *t* test with Bonferroni correction for multiple comparisons.

Endocytosis of fluorescently labeled transferrin (568-Tf) was utilized as a functional readout for CME. The kinetics of 568-Tf endocytosis was measured in HEK293T cells, transfected to express α-Syn or a mock pcDNA plasmid. Forty eight h post-DNA transfection cells were conditioned in serum-free Dulbecco's modified Eagle's medium (DMEM) for 90 minutes to enhance the localization of transferrin receptor at the plasma membrane. 568-Tf was applied for 0-12 min at 37 °C to allow binding and internalization of 568-Tf. Cells were then acid-washed to remove surface-bound 568-Tf and processed to visualize and quantify 568-Tf by confocal microscopy (Fig. 3*A*). A higher degree of 568-Tf endocytosis was detected in α-Syn over-expressing cells compared with the mock expressing cells. The significant differences were observed starting from 3 min of incubation and furthermore at 7 and 12 min (Fig. 3*A*).

In a complement experiment, endogenous α-Syn expression was down-regulated in the SK-Mel2 cells using shSNCA and were 24% of the levels detected in control cells, infected with shCntrl (Figs. 1*C* and 3, *B* and *C*). α-Syn levels were kept down-regulated for at least 14 days and experiments were performed during this time window. Endocytosis of 568-Tf, following 7 min of incubation, was significantly (55%) lower in shSNCA than in shCntrl cells (set at 100%, Fig. 3, *B* and *C*). In agreement with our recent report (5), silencing α-Syn expression resulted in significantly lower levels of PI(4,5)P_2_ (73%) compared with control cells (set at 100%). Similarly, PI(3,4)P_2_ levels were also lower (66%) with silencing α-Syn expression (ICC).

To verify that the observed loss of PI(4,5)P_2_ was specific to the plasma membrane, we utilized the PH(PLCδ)-GFP biosensor for PI(4,5)P_2_ detection (47). SK-Mel2 cells expressing either shSNCA or shCntrl were transfected to express PH(PLCδ)-GFP. The signal ratio of GFP fluorescence in the plasma membrane to cytosolic GFP was calculated and used to indicate plasma membrane PI(4,5)P_2_. Importantly, the results obtained with the PH(PLCδ)-GFP biosensor were highly similar to the results with anti-PI(4,5)P_2_ antibody (Fig. 3, *D* and *E*) and confirmed the significant reduction in plasma membrane PI(4,5)P_2_ in α-Syn–depleted cells (*i.e.* 83% of PH(PLCδ)-GFP signal ratio compared with control cells, set at 100%).

To assess the general effects of α-Syn on PIPs, we utilized an inducible SH-SY5Y cell line, expressing α-Syn under the control of Dox (Fig. 3, *F* and *G*) (48). α-Syn expression was induced for 72 h and cells were processed for the detection of PIPs by FACS. Control cells that express a mock plasmid were treated in parallel. Significantly higher levels of PI4P, PI(3,4)P_2_, and PI(4,5)P_2_ were detected with inducing the expression of α-Syn compared with the control cells (set at 100%). In contrast, PI3P and PI(3,4),5P_3_ levels were not altered upon α-Syn overexpression (Fig. 3*G*).

These data suggest that α-Syn regulates the levels of PI(3,4)P_2_ and PI(4,5)P_2_ phosphoinositides that control CME of transferrin. We therefore decided to test the hypothesis that α-Syn increases PIP_2_ levels to enhance CME.

### α*-Syn-mediated endocytosis of transferrin is PI(4,5)P_2_-dependent*

To experimentally regulate the levels of PI(4,5)P_2_, we utilized an inducible enzymatic system to acutely deplete PI(4,5)P_2_ from the plasma membrane (49). This system enables rapamycin-induced targeting of Inp54p, a PI(4,5)P_2_-5-phosphatase, to the plasma membrane. HEK293T cells were transfected to co-express the inducible phosphatase together either with WT α-Syn or pcDNA mock plasmid. 48 h post-DNA transfection, cells were processed simultaneously for 568-Tf endocytosis together with activation of the phosphatase with rapamycin (see “Experimental procedures”).

**Figure 4:**
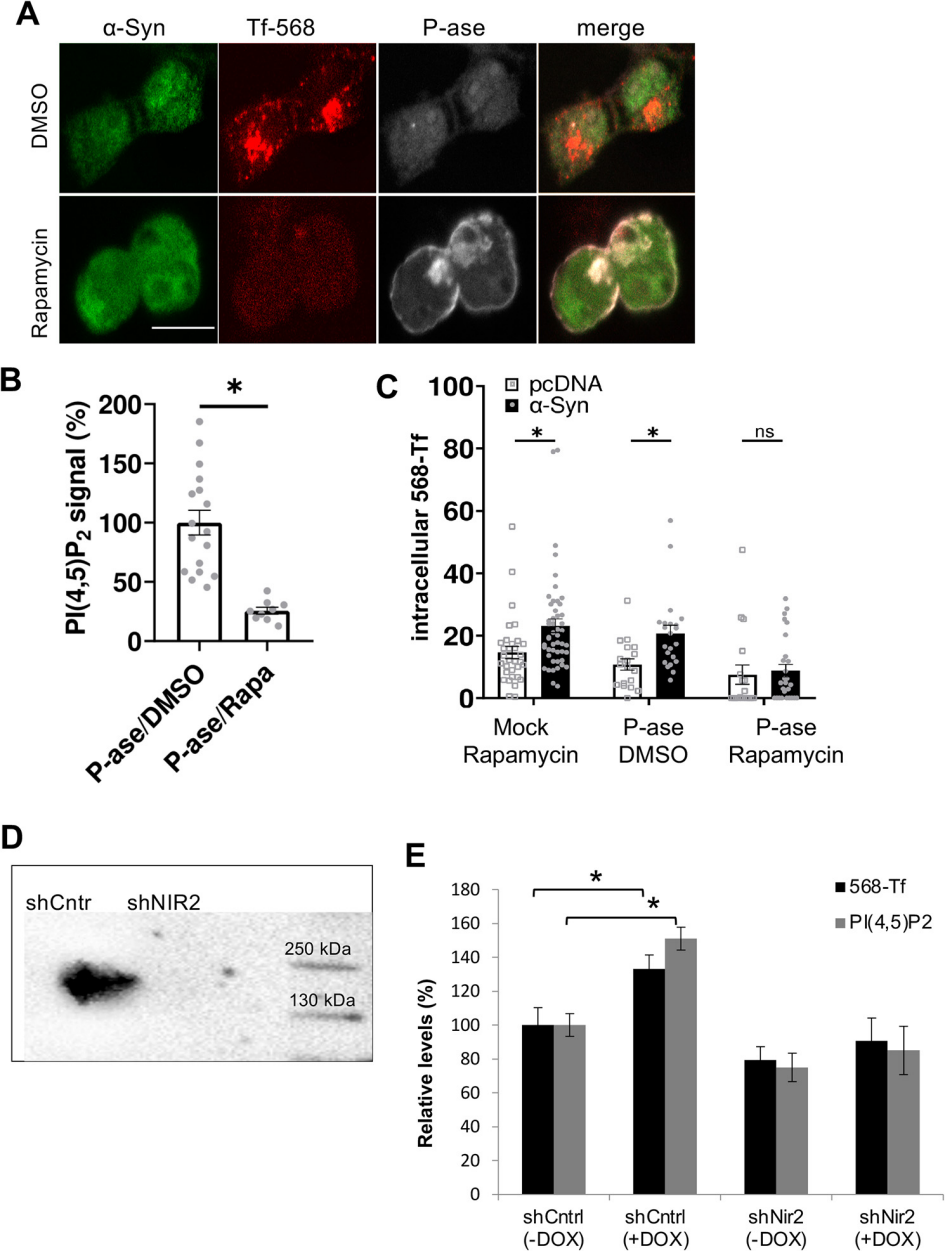
**The enhancing effect of** α**-Syn on CME is PI(4,5)P_2_ dependent.***A,* HEK293T cells, transfected to co-express the three plasmids: Lyn-FRB, FKBP-CFP-Inp54p, and WT α-Syn. On the day of the experiment, cells were serum starved for 90 min and then incubated for 7 min with 568-Tf and rapamycin (500 nm) to induce the recruitment of the Inp54p phosphatase (*P-ase*) to the plasma membrane along with internalization of 568-Tf. Control cells were treated in parallel with DMSO (0.5%, v/v). Cells were then acid-washed, fixed, and immunoreacted with anti-α-Syn ab (MJFR1; *green*). Direct fluorescence for CFP (P-ase; *gray*) and 568-Tf (*red*) is shown. *Bar* = 10 μm. *B,* HEK293T cells expressing Lyn-FRB and FKBP-CFP-Inp54p were treated either with rapamycin or DMSO as described in *A*, followed by immunoreaction with anti-PI(4,5)P_2_ ab (Echelon). A quantification of PI(4,5)P_2_ signal is shown (*n* > 9 cells per treatment; mean ± S.E.; *, *p* < 0.01 *t* test). *C,* graph showing quantification of internalized 568-Tf signal in cells transfected and treated as in *A*. Cells were transfected with a mock vector (*white bars*, *squares*) or α-Syn (*black bars*, *circles*). Mean ± S.E. Shown a representative experiment (out of *n* = 4 experiments); *n* > 18 cells per treatment in each experiment; *, *p* < 0.05 *t* test; with Bonferroni correction for multiple comparisons. *D,* the inducible Tet-on SH-SY5Y were infected with shNir2 or a control shRNA (*shCntrl*). Protein samples were analyzed by Western blotting and immunoreacted with anti-Nir2 ab (Abcam). *E,* cells as in *D* were induced to express α-Syn with Dox (1 µg/ml, for 72 h) or treated in parallel without Dox. Cells were then processed for ICC to measure 568-Tf endocytosis, as described in *C*. Mean ± S.E., 3 experiments, *n* = 20-25 cells per treatment; *, *p* <0.05, *t* test. Sister cultures were immunoreacted with anti-PI(4,5)P_2_ ab and analyzed by FACS. Mean ± S.E. of 3 experiments, each consisting of *n* > 2000 cells per sample; *, *p* <0.05 *t* test; with Bonferroni correction for multiple comparisons.

The Inp54p phosphatase is recruited to the plasma membrane in cells treated with rapamycin but remains in the cytoplasm in DMSO-treated cells (Fig. 4*A*). In accord, PI(4,5)P_2_ levels were lower in rapamycin (26%) compared with the DMSO-treated cells (set at 100%), demonstrating phosphatase activity (ICC; Fig. 4*B*). To find out whether plasma membrane PI(4,5)P_2_ levels play a role in α-Syn' effect to enhance endocytosis, we quantified 568-Tf internalization in cells that co-express Inp54p together with WT α-Syn and treated with rapamycin or DMSO (Fig. 4, *A* and *C*). The results show that rapamycin-induced depletion of PI(4,5)P_2_ completely abolished the ability of overexpressed α-Syn to stimulate CME (Fig. 4*C*), whereas CME was stimulated by α-Syn expression in cells treated with DMSO (vehicle), measuring a higher degree of 568-Tf internalization (193%) compared with the control cells that express a mock pcDNA (set at 100%). In control cultures, in which cells were transfected and treated in parallel but without Inp54p expression, we found no effect for rapamycin on α-Syn–induced CME (Fig. 4*C*).

To confirm a role of PI(4,5)P_2_ in α-Syn–mediated endocytosis of Tf-568, we tested the significance of silencing Nir2 expression. An important function of Nir2 protein is the exchange of endoplasmic reticulum PI with PM phosphatidic acid (PA), which is required for maintaining PM levels of PI(4,5)P_2_ (50). Nir2 expression was silenced with shNir2 in the inducible α-Syn expressing SH-SY5Y cell line, resulting in ∼70% lower Nir2 mRNA levels and lower protein levels of the respective levels detected in control cells, infected to express shCntrl (Fig. 4*D*). α-Syn expression was then induced with doxycycline for 72 h and cells were analyzed by FACS (*n* > 2000 cells) to detect PI(4,5)P_2_ and α-Syn levels. Sister cultures were analyzed by ICC (*n* = 20-25 cells) to detect internalized 568-Tf (Fig. 4*E*). Inducing α-Syn expression with doxycycline resulted in significantly higher PI(4,5)P_2_ (151%) levels and in accord, higher internalization of 568-Tf (133%), compared with cells that were not treated to induce the expression of α-Syn (100%). However, in cells that their Nir2 expression was silenced, induction of α-Syn expression had no effect on PI(4,5)P_2_ levels, nor endocytosis of 568-Tf. The results therefore suggest that interference with the homeostasis of PI(4,5)P_2_ on the PM inhibited the effects of α-Syn to enhance endocytosis of transferrin.

### α*-Syn mutations correlate endocytosis of transferrin with changes in plasma membrane levels of PI(4,5)P_2_*

**Figure 5:**
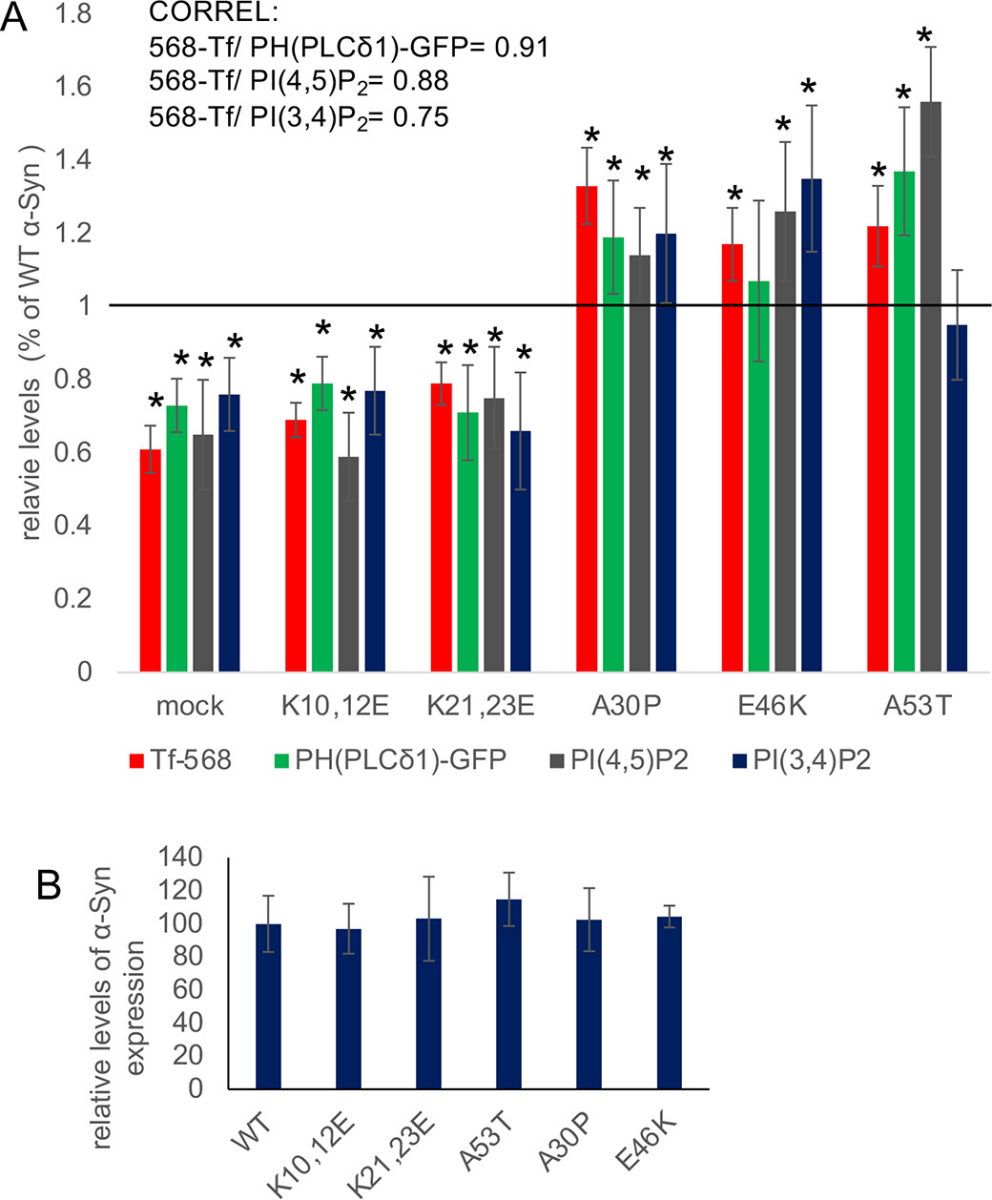
α**-Syn mutations correlate endocytosis of transferrin with changes in plasma membrane levels of PI(4,5)P_2_.***A,* HEK293T cells were transfected to express the indicated α-Syn forms. Cells were processed for 568-Tf endocytosis by ICC (*n* > 15 cells per treatment). Sister cultures were immunoreacted with anti-PI(4,5)P_2_ ab or anti-PI(3,4)P_2_ ab and analyzed by FACS. Plasma membrane PI(4,5)P_2_ was determined by calculating the membrane to cytosolic signal ratio of PH-PLCδ1-GFP in cells expressing WT α-Syn or the specified α-Syn mutations. *Vertical line* represents WT α-Syn, set at 100%. Mean ± S.E. of *n* = 3-4 experiments. In each experiment *n* = 20-25 cells (ICC) or >2000 cells (FACS) were used per treatment. *, *p* < 0.05, *t* test with Bonferroni correction. *CORREL*, correlation coefficient. *B,* cells as in *A* showing the α-Syn signal determined by FACS, presented as percent of the signal determined for WT α-Syn in each experiment. Mean ± S.D., *n* = 3-4 experiments, >2000 cells for each α-Syn construct.

Endocytosis of 568-Tf and PIP_2_ levels were determined in HEK293T cells, expressing either one of the following α-Syn forms, WT α-Syn; the PD-associated mutations in α-Syn, A30P, E46K or A53T; or the synthetic K10,12E or K21,23E mutations. The synthetic mutations in α-Syn were generated by replacing two positively charged lysine residues within the KTKEGV repeat domain, with negatively charged glutamic acid residues. In a previous study, these Lys to Glu mutations were shown to interfere with α-Syn binding to membrane phospholipids (51). α-Syn expression, PI(3,4)P_2_, and PI(4,5)P_2_ levels were determined by FACS, using specific abs (*n* < 2000 cells; Fig. 5, *A* and *B*) and plasma membrane levels of PI(4,5)P_2_ were determined by the PH(PLCδ)-GFP signal ratio (as above; *n* = 20-25 cells; Fig. 5*A*). The results show significantly higher levels of 568-Tf endocytosis, PI(3,4)P_2_, and PI(4,5)P_2_ in WT α-Syn than in the mock-plasmid expressing cells. Further increases over WT α-Syn were generally detected for these measured variables with the PD-associated mutations, with the exception of the A53T effect on PI(3,4)P_2_ and the E46K effect on plasma membrane PI(4,5)P_2_ levels. The levels determined in the Lys to Glu mutations in α-Syn were lower compared with WT α-Syn-expressing cells (Fig. 5*A*). Comparable levels of α-Syn expression were detected for the tested α-Syn constructs (Fig. 5*B*).

A strong correlation between α-Syn effects on 568-Tf endocytosis and plasma membrane levels of PI(4,5)P_2_ was noted (Fig. 5*A*; correlation coefficient (*r*) = 0.91). Similarly, 568-Tf endocytosis correlated with the total signal of PI(4,5)P_2_ (*r* = 0.88). 568-Tf endocytosis also correlated with PI(3,4)P_2_ levels (*r* = 0.75). We thus concluded that α-Syn increases PIP_2_ levels to facilitate CME and decided to test the hypothesis that it similarly acts to enhance SV endocytosis.

### α*-Syn accelerates the rate of SV endocytosis alongside with reducing the fraction of released SVs*

The involvement of α-Syn in SVs cycling was tested using Synaptophysin-2XpHluorin (sypHy) (52, 53). The pH-dependent fluorescence of sypHy, which is quenched in intact acidified SVs, increases upon exocytosis. After endocytosis and re-acidification of the SV lumen, fluorescence is re-quenched and returns to baseline. Primary hippocampal neurons prepared from α-Syn^−/−^ (C57BL/6JOlaHsd) mouse brains were infected to express sypHy together with one of the following α-Syn forms, WT α-Syn, the A30P, E46K, A53T mutants, or the two Lys to Glu mutations. mCherry served as a control for infection efficacy. SV cycling was measured at 13 days *in vitro* (DIV) by imaging sypHy before, during, and after the delivery of 300 stimuli at 20 Hz (13, 54). NH_4_Cl saline was applied following the return of fluorescence to baseline, to alkalinize all intracellular compartments, thus exposing the total size of the SV pool (*F*_max_).

**Figure 6:**
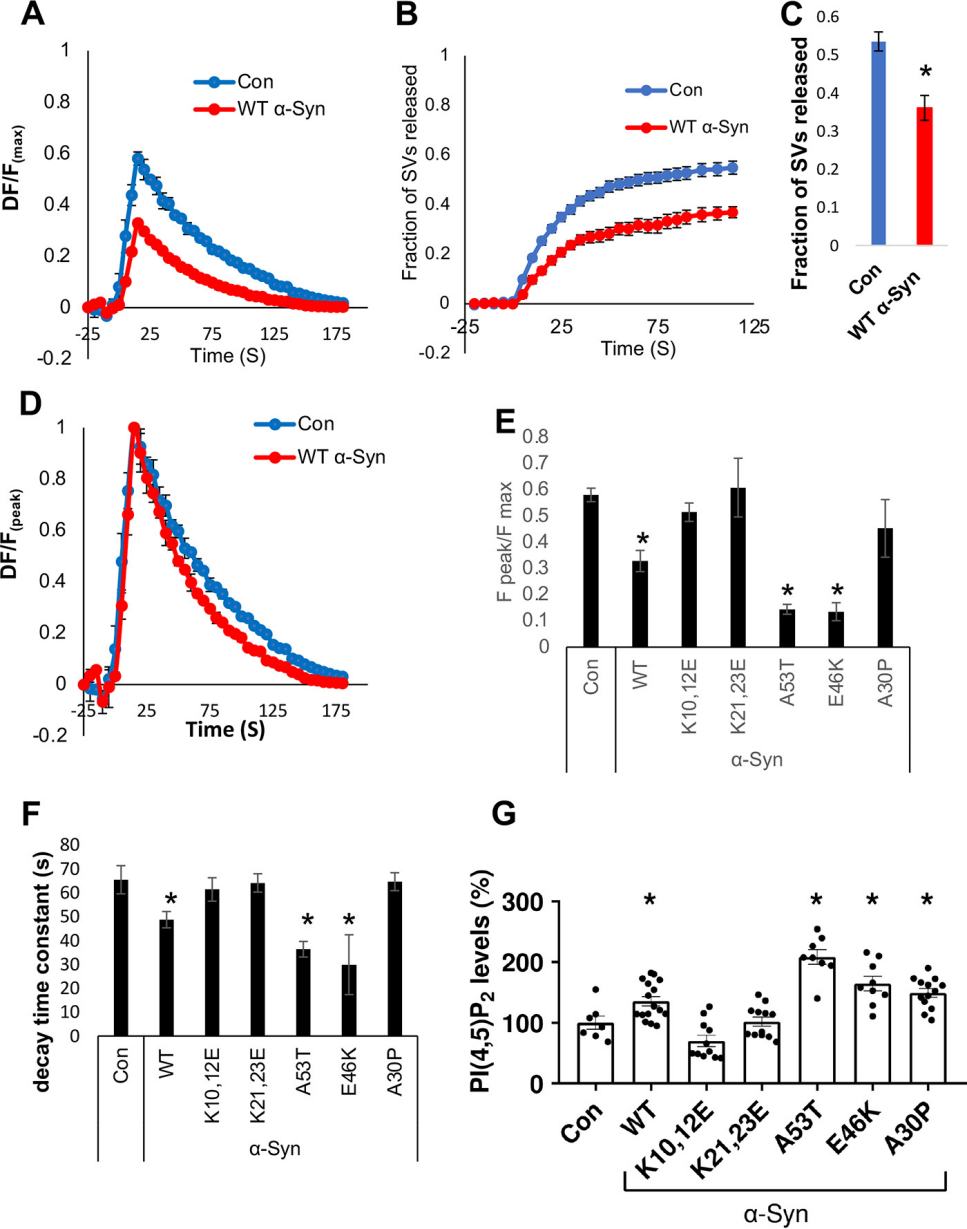
α**-Syn mutations differentially affects SV endocytosis and neuronal PI(4,5)P_2_ levels.***A,* hippocampal neurons at 13 DIV, prepared from α-Syn^−/−^ mouse brains were infected to express sypHy and mCherry, and either WT α-Syn or a mock plasmid. Neurons were stimulated for 15 s at 20 Hz (300 stimuli) at room temperature and the change in sypHy fluorescence in the synaptic puncta was recorded. The baseline fluorescence prior to stimulation (*F*_0_) was subtracted. Fluorescence was normalized to the total pool of vesicles (*F*_max_) measured at the end of the measurements by exposure to NH_4_Cl-saline. Mean ± S.E. of *n* = 6 slides per treatment (50 synapses per image, in 3 independent experiments). *B,* as in *A*, but the bath included 10 μm BafA, and 2400 stimuli were applied at 20 Hz. *C,* shown is the fluorescence measured 120 s after starting stimulation (as in *B*), normalized by the total pool of vesicles (*F*_max_), mean ± S.E.; *n* = 12-20 slides, 30-50 synapses per image). *p* < 0.001 *t* test. *D,* cells were described as in *A*. Shown is Δ*F* scaled to the peak fluorescence (*F*_peak_). Mean ± S.E.; *n* = 6 slides per treatment (50 synapses per image, in 3 independent experiments). *E*, fractional peak release values (*F*_peak_/*F*_max_) for each of the specified α-Syn forms. Shown are mean ± S.E. values; *n* = 3-6 slides per treatment (30-50 synapses per image, 3 experiments). *, *p* < 0.05 *t* test; with Bonferroni correction for multiple comparisons. *F,* graph showing the calculated decay constant resembling the rate of endocytosis with each of the specified α-Syn forms or the control plasmid. *n* = 3-6 slides per treatment (30-50 synapses per image, 3 experiments). *, *p* <0.05 *t* test; with Bonferroni correction for multiple comparisons. *G,* hippocampal neurons expressing the indicated α-Syn forms or a mock plasmid were processed for ICC at 13 DIV and immunoreacted with anti-α-Syn ab (MJFR1) and anti-PI(4,5)P_2_ ab (Echelon). Graph showing the quantification of PI(4,5)P_2_ in α-Syn positive neurites. Mean ± S.E.; *n* = 7-16 fields per treatment *, *p* < 0.05 *t* test; with Bonferroni correction for multiple comparisons.

The results show that WT α-Syn expression over the α-Syn^−/−^ background inhibits the extent of SV cycling, represented by a lower peak fluorescence (*F*_peak_/*F*_max_) (Fig. 6*A*; *n* = 50 synapses per image, 3 experiments). The lower peak level of the sypHy signal is in agreement with previous reports on an inhibitory role for α-Syn in exocytosis (11). A lower sypHy signal may arise either from a reduction in the number of SVs available for release, an acceleration of endocytosis or both. To assess specifically SV exocytosis, we added bafilomycin A (BafA) to the bath. BafA inhibits re-acidification of the SVs after endocytosis and thus, sypHy measurements performed in its presence report exclusively exocytosis (53). Indeed, in the presence of BafA, the cumulative sypHy signal in WT α-Syn expressing neurons was lower than in control neurons (Fig. 6, *B* and *C*), indicating a reduction in the total secretory capacity of the presynaptic terminals, as has been previously reported (54, 55). Importantly, normalizing the traces by the peak fluorescence obtained at the completion of stimulation (Δ*F*/*F*_peak_) revealed that the kinetics of the decay of sypHy was accelerated by the expression of WT α-Syn (Fig. 6, *D* and *F*). Thus, in addition to its inhibitory effect on the exocytotic segment of the SV cycle, α-Syn also accelerates the rate of endocytosis.

The PD-associated mutations in α-Syn, E46K, and A53T, further inhibited SV recycling (Fig. 6*E*) and in accord, further accelerated the rate of endocytosis (Fig. 6*F*). However, the A30P mutation and both Lys to Glu mutations were not different from control cells in their effects on SV recycling (Fig. 6, *E* and *F*). Together, measurements of α-Syn effects on SVs cycling, as determined by sypHy, reveal its complex effects on SV pools and architecture. However, considering the actual segment of SVs trafficking, α-Syn appears to accelerate the rate of endocytosis.

We next assessed PI(4,5)P_2_ levels in primary neurons infected to express WT α-Syn or the specified mutations as above. At 13 DIV, neurons were fixed and processed for ICC with anti-α-Syn and anti-PI(4,5)P_2_ antibodies. Similar to the results in HEK293T cells (Fig. 5), we found that expression of α-Syn mutations in hippocampal neurons differentially affected PI(4,5)P_2_ levels (Fig. 6*G*). That is, WT α-Syn increased PI(4,5)P_2_ levels (136%) over the levels detected in control cells (set at 100%); the PD-associated A30P, E46K, and A53T mutations further increased PI(4,5)P_2_ levels (150–208%), however, PI(4,5)P_2_ levels in primary neurons expressing the Lys to Glu mutations did not differ from control cells.

The results further demonstrate a correlation between α-Syn-dependent increases in PI(4,5)P_2_ levels and its capacity to enhance the rate of endocytosis. That is, an inverse correlation of *r* = −0.75 was calculated between the decay-constant of sypHy signal and PI(4,5)P_2_ levels with the different α-Syn mutations. Excluding the A30P mutation, which appears ineffective in SV endocytosis, yet increases PI(4,5)P_2_ levels, results in a stronger correlation (*r* = −0.87).

**Figure 7:**
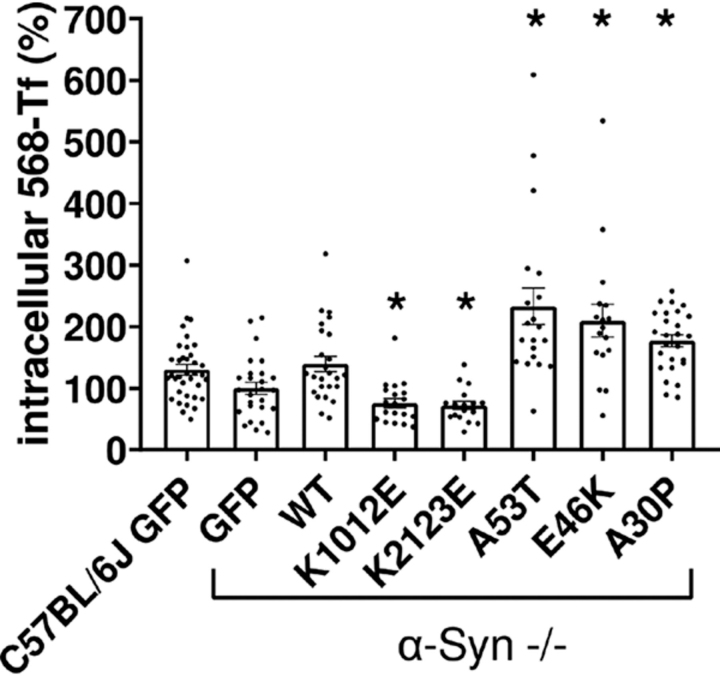
**CME in primary neurons.** Primary neurons from WT mice (C57BL/6J) or α-Syn^−/−^ (C57BL/6JOlaHsd) mice were transfected to express the indicated α-Syn forms or a mock GFP plasmid. At 8 DIV cells were starved for 90 min, incubated for 7 min with 568-Tf, and then acid washed. Cells were immunoreacted with anti α-Syn ab (Syn211). The signal obtained for Tf-568 in neuronal somas was quantified. *n* = 17-36 cell somas per treatment *, *p* <0.05 *t* test; with Bonferroni correction for multiple comparisons.

To further assess the effects of A30P mutation in CME, in neuronal cells, we next determined 568-TF endocytosis in primary cortical neurons prepared from WT (C57BL/6J) or α-Syn^−/−^ (C57BL/6JOlaHsd) mouse brains. α-Syn^−/−^ neurons were infected to express WT α-Syn, the specified mutations as above, or a mock GFP plasmid. Neurons obtained from the WT mouse brains were also infected to express the GFP plasmid. At 8 DIV, neurons were conditioned with B27-free medium for 90 min, 568-Tf was added to the cells for 7 min at 37 °C, cells were then acid-washed to remove surface-bound 568-Tf and fixed to determine direct fluorescence by confocal microscopy (Fig. 7). The result show highly similar levels of 568-Tf endocytosis in cortical neurons from C57BL/6J brains and α-Syn^−/−^ neurons infected to express WT α-Syn. Suggesting that the expression levels of α-Syn in the rescued α-Syn^−/−^ neurons and the WT C57BL/6J neurons are closely similar. WT α-Syn expression enhanced endocytosis (139%) over the levels detected in control α-Syn^−/−^ neurons expressing the mock GFP plasmid (set at 100%); further increases in endocytosis were detected for the PD-associated A30P, E46K, and A53T mutations (177, 210, and 233%, respectively). Whereas the Lys to Glu mutations in α-Syn abolished the enhancing effects on endocytosis. All mutant α-Syn tested differed significantly from WT α-Syn in their effects on 568-Tf endocytosis. Thus, A30P mutation appears to effectively enhance endocytosis of 568-Tf and CME (Fig. 7). Yet, it interferes with α-Syn effects to enhance SV endocytosis at the synapse (Fig. 6).

## Discussion

We address the controversy over α-Syn's role in membrane trafficking and SV cycling by investigating α-Syn associations with PIP_2_ and specifically with PI(4,5)P_2_. We show that α-Syn colocalizes with components of clathrin-coated pits/vesicles, *e.g.* pAP2, PI(4,5)P_2_, and PI(3,4)P_2_. We further show that α-Syn–mediated CME requires PI(4,5)P_2_ at the plasma membrane. Utilizing a recruitable 5-phosphatase, which hydrolyses PI(4,5)P_2_ at the plasma membrane, we demonstrate that α-Syn–mediated CME is PI(4,5)P_2_-dependent. In addition, lowering PI(4,5)P_2_ levels by means of silencing the PI-transfer protein, Nir2, abolished the enhancing effect of α-Syn on 568-Tf endocytosis. Finally, utilizing specific α-Syn mutations, with differential effects on PI(4,5)P_2_ levels, we demonstrate a correlation between neuronal PI(4,5)P_2_ levels and the rate of SVs endocytosis, assessed by acidification of sypHy. Based on these results and the established role of PI(4,5)P_2_ in CME (29, 32, 56, 57, 58, 59), we conclude that α-Syn facilitates endocytosis by enriching the plasma membrane with PI(4,5)P_2_.

In line with previous reports (17, 54, 55), the results show that WT α-Syn expression inhibits the overall extent of SVs cycling, determined by the sypHy signal. This inhibition may result from attenuated mobility of the recycling pool of SVs in presynaptic boutons and/or reduced pool size at synapses. We interpreted the lower peak level of the sypHy signal, detected in α-Syn expressing neurons, to represent an acceleration in the rate of endocytosis. However, our results also confirm previous observations indicating a reduction in the total secretory capacity of the presynaptic terminals (17, 22, 54, 55), which may result from changes in SV pool size, structural and/or ultrastructural changes in the synapse (60). Overall, the rate of SVs endocytosis in the tested α-Syn forms, represented by the decay in sypHy signal, correlate with their effects on PI(4,5)P_2_ levels at the synapse. However, the A30P mutation in α-Syn is exceptional in this sense, it increases PIP_2_ levels, yet ineffective in SV cycling. In line with this result, the A30P has been reported in previous studies to differ to some extent from other PD-associated α-Syn mutations. For example, it was reported that the A30P mutation disrupts pre-synaptic localization of α-Syn (61) and disrupts α-Syn enrichment at synapses (61, 62). The result herein, showing that A30P α-Syn mutation enhances 568-TF endocytosis in neurons suggests that this mutation in α-Syn does not interfere with its effect on CME but with its synaptic activity.

There is a general agreement in the field concerning two features of α-Syn protein, its preference for binding acidic phospholipids (1) and its preference for curved membranes, akin the curvature that typifies synaptic vesicles (63). Thus, the findings herein, indicating that α-Syn interacts with and regulates PI(4,5)P_2_ levels, fit well with these two features. PI(4,5)P_2_ is an acidic phospholipid enriched on presynaptic membranes and due to its enrichment with PUFAs, helps to form membrane curvature (46). PI(4,5)P_2_ is critical for both mechanisms, endocytosis and exocytosis. Thus, recruitment of PIP_2_ to one mechanism will inevitably affect the other. Together with our previous findings, indicating a role for α-Syn to enrich membrane phospholipids with PUFA and increase membrane fluidity (9), it appears that α-Syn plays major roles in shaping membrane content and in accord, membrane function.

Cumulative data show that α-Syn colocalizes and interacts with components of the clathrin-coated pit. The results herein show that α-Syn interacts with phosphorylated AP2, PI(4,5)P_2_, and PI(3,4)P_2_ (Figure 1, Figure 2). It was shown to interact with clathrin (12, 13) and accumulate on CCPs that are arrested at the fission step in synapses of dynamin 1, 3 knocked-out neurons (13, 64, 65); α-Syn was also shown to interact with HSC70, which is recruited to the vesicle to facilitate vesicle uncoating (15). Together, these findings suggest that unlike several other endocytic proteins, α-Syn colocalizes with the CCP from its initiation throughout its maturation stages to fission and uncoating. Our data suggest that α-Syn acts to facilitate and increase the rate of transferrin and SV endocytosis. This finding is in line with our previous report (12) as well as other reports (13, 60). Of note, an inhibitory role for α-Syn in endocytosis was suggested in model systems that consist of excess of α-Syn, added directly to the synapse (4, 14, 15). In these studies, the excess of α-Syn appears to interfere with the process, either sequestering critical components in CME or shifting a delicate balance that is required for this highly orchestrated mechanism. Based on the data related to α-Syn interactions with PIPs, it is potentially possible that excess of α-Syn may alter the spatial and temporal balance of PIPs in the process.

An emerging question in α-Syn's involvement in mechanisms of membrane trafficking is: why do different studies report different outcomes and how can we get better consistency? To be able to solve this problem we may need to consider the following: 1) the type of endocytic mechanism; 2) neuronal activity; 3) α-Syn expression; and 4) the lipid content at the plasma membrane. Although CME is a key mechanism of SV endocytosis, additional routes of SV endocytosis, including, kiss-and-run, ultrafast, and bulk endocytosis take place at the synapse (66, 67). The degree of involvement and relative importance of each of these mechanisms during physiological neuronal function and in different neuronal types is not fully clear. It is possible that different neurons rely on different mechanisms of endocytosis, depending on their electrophysiological activity and the accompanied need in vesicle recycling (66, 67). In relevance to neuronal activity, it was suggested that the role α-Syn in endocytosis may differ between basal and intense neurotransmission (11). Moreover, neural activity has been shown to control the synaptic accumulation of α-Syn (68). Thus, the type of α-Syn expression model may affect the outcome, whether α-Syn^−/−^; α-, β-, γ-Syn^−/−^; stable (long term) or transient α-Syn over-expression; exogenously added or endogenously expressed α-Syn. α-Syn is a highly dynamic protein that responds to changes in its environment with structural changes that may affect its activity. Due to its multifaceted nature, it is not possible to consider an opposite outcome when comparing the results obtained in α-Syn silencing *versus* α-Syn over-expressing models. The results herein indicate that α-Syn actively regulates plasma membrane levels of PI(4,5)P_2_ to stimulate CME. Considering the above and the additional cellular mechanisms that rely on PI(4,5)P_2_ levels, it is important to also take PIPs homeostasis into consideration when analyzing α-Syn effects in membrane trafficking.

Abnormal homeostasis of PIPs links defects in membrane trafficking with neurodegeneration (69, 70). Mutations in Synaptojanin-1 (SynJ1), a PIP-phosphatase enriched in the brain, either in its Sac domain (R258Q and R459P), which dephosphorylates PI4P and PI3P to PI, or in the 5-phosphatase domain (Y832C), which dephosphorylates PI(4,5)P_2_ to PI4P, have been associated with early onset and typical PD (71, 72, 73, 74). Mice modeling loss of SynJ1 function, either carrying a PD-causing mutation (R258Q) or by haploinsufficiency (SynJ1^+/−^), demonstrate evidence for degeneration of the nigrostriatal dopaminergic system and accumulation of α-Syn pathology (75, 76). The alterations in PIPs homeostasis resulting from loss of SynJ1 activity has been associated with impaired autophagy, endocytic disfunction, and axonal damage (75, 76). Of relevance, in a recent report, we linked α-Syn' physiological activity in PI(4,5)P_2_ homeostasis with regulation of axonal plasticity and arborization. We further described evidence for a pathogenic role for α-Syn in dysregulating PI(4,5)P_2_ in PD (5). Here we extend these findings to show that α-Syn–mediated alterations in PIPs are also involved in its regulation of SV recycling and CME.

## Experimental procedures

### Plasmids

WT α-Syn or the specified α-Syn mutations were cloned into a pcDNA vector (51). Costume-ready Mission shNIR2 (TRCN0000029763), shSNCA (TRCN0000272292), and shCntrl were from Sigma-Aldrich (Rehovot, Israel) (5). The following plasmids were used: pGFP-C1-PLCδ1-PH (Addgene number 21179 from Tobias Meyer (47)), CFP-FKBP-Ins54p and Lyn-FRB (49), BFP2-INPP4B-CAAX (77), and INPP5E-mCherry (34). In addition, an adenovirus expressing sypHy under the human synapsin (hSyn) promotor (AAV1/2 hSyn:Synaptophysin-2XpHluorin) and AAV1/2 hSyn:WT α-Syn (54) were used. AAV1/2 hSyn:mutants α-Syn were constructed by PCR amplification followed by complete sequencing of the inserts.

### Mice

The human PrP-A53T α-Syn tg (78), α-Syn^−/−^ C57BL/6JOlaHsd mouse lines (79) or WT C57BL/6J mice were used. All animal welfare and experimental protocols were approved by the Committee for the Ethics of Animal Experiments of the Hebrew University of Jerusalem NIH approval number OPRR-A01-5011 (permit number: MD-16-14826-3).

Mice were housed at a 12-h dark/light cycle and were allowed free access to food and water. This study was carried out in strict accordance with the recommendations in the Guide for the Care and Use of Laboratory Animals of the National Institutes of Health. Adequate measures were taken to minimize pain and suffering.

### Cells

HEK293T, HeLa, and an inducible α-Syn–expressing SH-SY5Y cell line (48) were maintained in DMEM supplemented with 10% FBS, 2% l-glutamine, 1% penicillin/streptomycin, sodium pyruvate, and nonessential amino acids (Biological Industries, Beit-Haemek, Israel). α-Syn expression was induced in the inducible SH-SY5Y cell line with 1 μg/ml of doxycycline (Dox, Sigma-Aldrich, Rehovot, Israel). SK-Mel2 cells express detectable levels of endogenous α-Syn, however, these are lowered with passages. Thus, a large number of aliquots at passage 12 were kept frozen and experiments were performed between weeks 2 and 6 from thawing a frozen aliquot. SK-Mel2 cells were maintained in minimum essential medium (Sigma-Aldrich, Rehovot, Israel) supplemented with 10% FBS, 1% l-glutamine, penicillin/streptomycin, and sodium-pyruvate. Cultures were maintained at 37 °C in a 95% air, 5% CO_2_ humidified incubator.

### Primary cultures

Primary hippocampal cultures were prepared by dissecting both hippocampi of P0-P1 C57BL/6JOlaHsd (α-Syn^−/−^) mouse brains, as described previously (80, 81). After trituration in 20 units/ml of papain solution (Worthington Lakewood, NJ, USA), 1 × 10^5^ cells were plated on coverslips that were pre-coated with 5 μg/ml of poly-D-lysine (Sigma-Aldrich, Rehovot, Israel) in a 24-well–plate containing 1.5 ml of Neurobasal-A medium (Gibco, ThermoFisher Scientific, Petah Tikva, Israel) supplemented with 5% fetal bovine serum, 2% B-27 (Gibco, ThermoFisher Scientific), 1% Glutamax I, and 1 μg/ml of gentamicin. After 1 day, the solution was replaced to 1 ml of Neurobasal-A supplemented with 2% B-27 and 1% Glutamax I. To slow the proliferation of glial cells, 1 μm cytosine β-d-arabinofuranoside (Ara-C; Sigma-Aldrich) was added to the culture at 2 DIV. Cultures were maintained at 37 °C in a 5% CO_2_ humidified incubator until used at 8-13 DIV as indicated. For measurements of Tf-568 endocytosis, neurons were prepared and grown as previously describe (5).

### sypHy imaging

Neurons were infected at 5 DIV with AAV1/2 hSyn:sypHy and were imaged at 13 DIV. Coverslips were placed in a field stimulation chamber (Warner Scientific, Hamden, CT, USA) in an extracellular solution composed of the following (in mm): 150 NaCl, 3 KCl, 20 glucose, 10 HEPES, 2 CaCl_2_, 2 MgCl_2_, pH adjusted to 7.35 with NaOH at 310 mOsm. The solution also contained glutamate receptor antagonists APV (50 μm) and DNQX (10 μm) to avoid recurrent network activity. Neurons were imaged at room temperature every 6 s on a Nikon TiE-inverted microscope equipped with a Neo5.5 Andor sCMOS camera, using an EGFP filter set (Chroma, Bellows Falls, VT, USA). After acquiring 6 baseline images (*F*_0_), neurons were stimulated by applying 300 bipolar pulses at 20 Hz, each of 1 ms duration and 10 V/cm amplitude, through parallel platinum wires. At the completion of the experiment, the culture was exposed to saline in which 50 mm NaCl_2_ was replaced with NH_4_Cl, to expose the total pool of vesicles (*F*_max_ (54)). The background-corrected fluorescence values recorded for each synapse were normalized either by the peak response during the stimulation train, or by the size of the total pool of vesicles, as indicated. The rate of endocytosis was assessed by exponential fitting of the time course of the decay in fluorescence from its peak upon the completion of stimulation back to baseline values. To exclusively image exocytosis, we added 1 μm BafA to the extracellular solution. BafA blocks the vesicle proton pump, thus masking the endocytotic segment of the SV cycle (53) without affecting the kinetics of endocytosis (82). Quantification was performed with NIS-Elements software (Nikon), by placing equal circular regions of interests (ROI) on 30-50 synapses in each field and extracting the background-subtracted average fluorescence value of each ROI (54). A local background was obtained adjacently to each ROI.

### Viral production and transduction

AAV1/2 particles were produced as previously described (81). Briefly, HEK293T cells were co-transfected with the pD1 and pD2 helper plasmids and a plasmid containing the cDNA of interest located between AAV2 ITRs, preceded by the hSyn promotor. After 3 days of incubation at 37 °C in a humidified 5% CO_2_ incubator, cells were lysed in lysis solution (150 mm NaCl, 50 mm Tris-HCl, pH 8.5) using 3 rapid freeze-thaw cycles (in an ethanol bath chilled to −80 °C and a heated 37 °C water bath). The supernatant was treated with 10 units/ml of benzonaze (Sigma-Aldrich, Rehovot, Israel), cleared by centrifugation, and filtered through a 0.45-μm membrane. The viral particles were maintained at 4 °C until use. Viral titer was determined by infecting neuronal cultures, aiming for 80–90% infection efficiency, verified by immunofluorescence or direct fluorescence imaging, as applicable. Viral titer was determined by adding 0.2-2 μl of the viral prep directly to the growth medium at 5 DIV.

Lentiviral particles were produced as described (5) by co-transfecting HEK293T cells with a set of three plasmids: pCMVΔR8.91, pMD2.G, and a transfer plasmid pLKO-1-puro. Virus titer was determined for each preparation following transduction of cells, by quantitative PCR using specific primers for the puromycin resistance gene: forward, 5′-TCACCGAGCTGCAAGAACTCT-3′ and reverse primer, 5′-CCCACACCTTGCCGATGT-3′; primer sequence for human SNCA, forward, 5′-GCAGGGAGCATTGCAGCAGC-3′ and reverse, 5′-GGCTTCAGGTTCGTAGTCTTG-3′; Nir2 forward, 5′-GCTTTGATGCACTCTGCCAC-3′ and reverse, 5′-AGCTCATTGTTCATGCTCCC-3′; G6PD forward, 5′-CACCATCTGGTGGCTGTTC-3′ and reverse 5′-TCACTCTGTTTGCGGATGTC-3′. SK-Mel2 and SH-SY5Y cells were infected by incubating the cells (1 × 10^6^) in FBS-free DMEM, containing viral particles and Polybrene (4 μg/ml) for 6 h. The conditioning medium was then replaced with 10% FBS-supplemented DMEM.

### FACS

Analysis was performed as previously described (5). Briefly, cells were fixed in 2% paraformaldehyde at 4 °C and permeabilized in 0.2% saponin in 1% BSA (w/v) for 15 min at 4 °C. Cells were then incubated with anti-α-Syn antibody (MJFR1, 1:2,000 Abcam, Zotal, Tel Aviv, Israel) and anti-PIP abs (Echelon Biosciences, Salt Lake City, UT), including: PI3P (Z-P003; 1:300), PI4P (Z-P004 1:300), PI(3,4)P_2_ (Z-P034b; 1:300), PI(4,5)P_2_ (Z-P045 1:200; Z-A045 1:800), and PI(3,4,5)P_3_ (Z-P345b; 1:400), for 90 min with gentle rolling; washed and probed with the respective secondary antibody for 30 min at room temperature. Analyses were performed using BD LSRFortessa Cell Analyzer, equipped with 5 lasers (355, 405, 488, 561, and 640 nm) and the FLOWJO, LLC software. Each experiment also included relevant compensation controls. A control consisting of cells grown and processed in parallel, treated with 10 μm ionomycin (46) for 5-7 min at room temperature, was also included. Gating was based on FSC, SSC, and positive immunoreactivity for α-Syn. A total of 2000-4000 gated cells were counted in each experiment unless indicated differently. Lentiviral particles were prepared as previously (5).

### Transferrin endocytosis

Measurement of transferrin endocytosis were performed as previously described (12, 34) with some modifications. Cells were grown in 12-well–plates, on cover slides that were pre-treated with poly-D-lysine (100 μg/ml) for 1 h. On the day of the experiment, cells were serum-starved for 1.5 h, or treated with media lacking B27 supplementation (primary neurons). Cells were then conditioned in 25 μg/ml of 568-Tf (Molecular Probes, Invitrogen, Rhenium, Israel) in clear DMEM at 37 °C for the time indicated. When specified, induction of FRB-FKBP dimerization and recruitment of Inp54p to the plasma membrane was achieved with the addition of rapamycin (500 nm) in DMSO (0.5% v/v). After two washes with ice-cold PBS, cells were acid washed at pH 5.3 (0.2 m sodium acetate, 0.2 m sodium chloride) on ice for 1.5 min, to remove surface-bound transferrin. Cells were then washed 2 additional times with ice-cold PBS, fixed in 2% paraformaldehyde for 20 min on ice, and processed for ICC.

### PI(4,5)P_2_ detection by the PH-PLCδ1-GFP biosensor

Cells were grown in 12-well–plates, on cover slides that were pre-coated with poly-d-lysine (100 μg/ml, for 1 h). Cells were transfected to express PH-PLCδ1-GFP using Jet-PRIME transfection reagent (Polyplus, France). When indicated, cells were co-transfected with WT α-Syn or one of the specified α-Syn mutations (A30P, E46K, A53T, K10,12E, or K21,23E), or a mock plasmid. In some experiments, cells were conditioned in the presence of 50 μg/ml of 647 concanavalin A (ConA, molecular probes, Invitrogen, Rehovot, Israel) in DMEM, at 37 °C for 10 min to label the plasma membrane. Membranes were defined by the ring-shaped ConA signal around the cell and were differentiated from the cytoplasm. Membrane to cytosolic PH-PLCδ1-GFP signal ratio was calculated using the NIS-Element AR Analysis 4.20.02 64-bit software (Nikon, Agentek, Tel Aviv, Israel) (5).

### ICC

Cell lines or primary neuronal cultures were fixed in cold 2% paraformaldehyde for 20 min, washed in PBS, and permeabilized with 0.5% saponin in blocking solution (1% BSA in PBS (w/v)) for 30 min at room temperature. Cells were incubated with anti-α-Syn antibodies, Syn211 (at 1:750 dilution, overnight at 4 °C); MJFR-1 (1:2000), LB509 (ab27766; 1:250), or anti-α-Syn ab21976 (1:330) from Abcam, Zotal, Tel Aviv, Israel. The immunoreactive pattern of these anti-α-Syn antibodies in SK-Mel2 cells is shown in Fig. S1 (P-AP2M1-T156 (D4F3; Cell Signaling Technology; 1:300). The following antibodies were from Echelon Biosciences (Salt Lake City, UT, USA): anti-PI(3,4)P_2_ (Z-P034b; 1:300) or anti-PI(4,5)P_2_ (Z-P045 1:200; Z-A045 1:800) for 2 h at room temperature. Cells were then washed (PBS; 10 min ×3) and incubated with a host-suitable secondary ab, washed again, and mounted in Vectashield mounting medium (Vector Laboratory, Burlingame, CA USA).

### Immunohistochemistry

Paraffin-embedded, coronal mouse brain sections (6 μm) were processed for immunostaining as previously described (5). Antigen retrieval was performed by heating the slides to 95 °C for 12 min in a pressure cooker. Slides were then permeabilized with 0.1% Triton in PBS (v/v) for 10 min, blocked with CAS-BLOCK solution for 10 min (ThermoFisher), and reacted with primary antibodies diluted in 1% BSA for 2 h. The following abs were used: anti-α-Syn (ab21976; 1:200), anti PI(3,4)P_2_ (1:200), anti-PI(4,5)P_2_ (Z-A045 1:400), and anti-pAP2 (P-AP2M1-T156, 1:200). Slides were washed and incubated with the host-matching secondary antibodies. Images were captured using Nikon's A1R+ confocal microscope, as described below.

### Fluorescence microscopy and image analysis

Images were acquired using a Zeiss LSM 710 Axio Observer confocal Z1 laser scanning microscope, equipped with an argon laser 488, Diode 405-430 laser and HeNe 633 laser.

For colocalization analyses, images were captured using Nikon's A1R+ confocal microscope, equipped with an ultrahigh-speed resonant scanner and high-resolution digital galvano scanner, with four laser unit LU-N4S. Per each experiment, the exciting laser, intensity, background levels, photomultiplier tube gain, contrast, and electronic zoom were maintained constant. The antibody-specific background was subtracted. The focus of each picture was obtained by choosing the plane with greatest fluorescent signal. Quantifications were performed with NIS-element software. A constant threshold for the signal of α-Syn and each PIP was maintained to all images. The nucleus was excluded from quantification. The program automatically defined the positive spots for either α-Syn or each PIP. Then, it automatically calculated the number of positive pixels in colocalization for each channel. Results were normalized to total α-Syn–positive pixels. Quantifications of total cellular signal of 568-Tf, α-Syn, or PIP_2_ levels were performed using ImageJ software.

### Western blotting

Samples of lysed cells (70 μg of protein) were loaded on a 10% SDS-PAGE and following electrophoresis were transferred to a polyvinylidene difluoride membrane. Membrane was blocked with milk (10% in TBST) and reacted with anti-Nir2 ab (ab22823, Abcam, 1:500). The immunoblots were reacted with a secondary, horseradish peroxidase-conjugated, anti-goat antibody (1:100,000, Jackson ImmunoResearch), visualized with Clarity Western ECL Substrate (Bio-Rad, Rishon Le Zion, Israel), and scanned with ChemiDoc XRS+ imaging system (Bio-Rad). Signal density was quantified using UN-SCAN-IT GEL 3.1 software (Silk Scientific, Orem, UT USA).

### Experimental design and statistical analysis

All experiments were performed in parallel with their designed controls and in random order, and they were replicated at least three times. Data are shown as mean ± S.E. Statistical comparisons were performed with the two-tailed Student's *t* test. When multiple comparisons were performed, we applied the Bonferroni correction. The distribution of the variables in each experimental group was within a normal range. All tests were conducted using GraphPad Prism version 8.0.1. Significant differences were accepted at *p* < 0.05.

## Data availability

All data presented are contained within the manuscript
