# Supplementary material for: α-Synuclein facilitates endocytosis by elevating the steady-state levels of phosphatidylinositol 4,5-bisphosphate
Source: J Biol Chem. 2021 Jan 13;295(52):18076–90. doi: 10.1074/jbc.RA120.015319 (PMC7939461; doi:10.1074/jbc.RA120.015319)
Supplement: Supplementary file 1 [file mmc1.pdf]

Supplementary Fig. 1

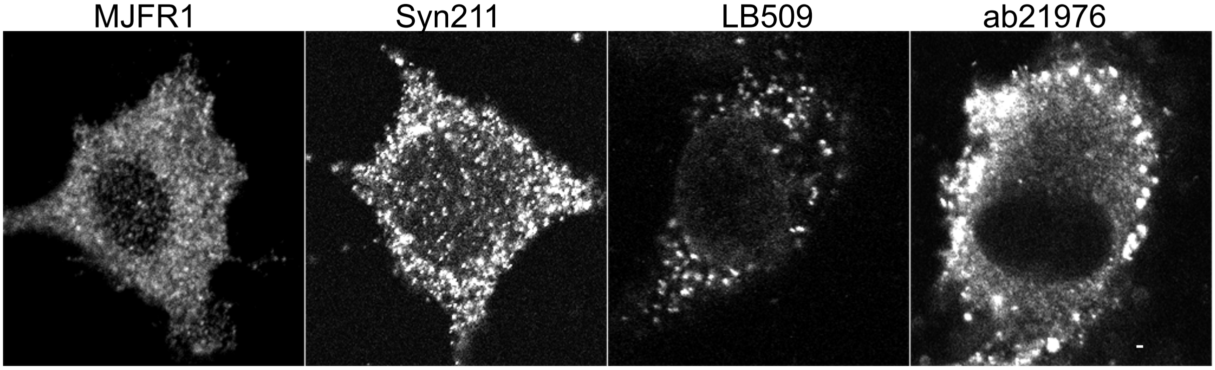

Supplementary Fig. 1. Anti α-Syn antibodies reacted with endogenous α-Syn in SK-Mel2 cells. Cells were incubated with the following anti α-Syn antibodies: MJFR1 (1:2000), Syn211 (1:750), LB509 (1:250) and ab21976 (1:330). Bar =10µm.
